# Development and validation of a search strategy and an automated classifier for retrieving temporomandibular disorders studies

**DOI:** 10.22514/jofph.2024.015

**Published:** 2024-06-12

**Authors:** Vicente Wielandt, Juan Fernando Oyarzo, Manolis Jusakos, Giuliana Lunecke, Diana Biscay, Claudia Bosio, Paula Zambrano-Achig, Sebastián Pinto, Magdalena Bignon, Gabriel Rada, Francisca Verdugo-Paiva

**Affiliations:** Orofacial Pain & TMD Program, Faculty of Odontology, Andres Bello University, 8370133 Santiago, Chile; Epistemonikos Foundation, 7550296 Santiago, Chile; Department of Paediatrics, Obstetrics & Gynaecology and Preventive Medicine and Public Health at the Autonomous University of Barcelona, 08193 Barcelona, Spain

**Keywords:** Temporomandibular disorders, Research methodology, Evidence-based dentistry, Systematic review, Automated classification

## Abstract

The objective was to develop and evaluate a comprehensive search strategy (SS) and automated classifier (AC) for retrieving temporomandibular disorders (TMD) research articles. An initial version of SS and AC was created by compiling terms from various sources, including previous systematic reviews (SRs) and consulting with TMD specialists. Performance was assessed using the relative recall (RR) method against a sample of all the primary studies (PS) included in 100 TMD-related SRs, with RR calculated for both SS and AC based on their ability to capture/classify TMD PSs. Adjustments were made iteratively. A validation was performed against PSs included in all TMD-relevant SRs published from January to April 2023. The analysis included 1271 PSs from 100 SRs published between 2002–2022. The initial SS had a relative recall of 89.34%, while the AC detected 70.05% of the studies. After adjustments, the fifth version reached 99.5% and 89.5% relative recall, respectively. Validation with 28 SRs from 2023 showed a search strategy sensitivity of 99.67% and AC sensitivity of 88.04%. In conclusion, the proposed SS demonstrated excellent performance in retrieving TMD-related research articles, with only a small percentage not correctly classified by the AC. The SS can effectively support evidence synthesis related to TMD, while the AC can aid in creating an open-access, continuously updated digital repository for all relevant TMD evidence.

## 1. Introduction

An updated, high-quality, and unbiased evidence synthesis is one of the most valuable contributions a research group can offer to stakeholders, including researchers, clinicians, guideline developers and policymakers. As systematic reviews (SRs) have been considered the standard for decision-making in health, the number of SR articles has risen substantially in the last decade. In 2010, a landmark study estimated that 11 SRs were published daily [1], and a recent study estimated that more than 100 SRs relevant to health decisions are released daily [2]. A comparable pattern has been reported in the oral health field, but studies highlighted that the increased volume of SRs may not reflect a steady improvement in the quality of the methods used in the published SRs [3, 4].

A critical step in conducting a high-quality SR is identifying all the relevant primary studies available through comprehensive searches. This process needs robust and validated search strategies (SS) to minimize bias and maximize the comprehensiveness of the evidence synthesis products, such as SRs, and clinical practice guidelines, among others. The SS validation process involves rigorously testing and refining search terms to identify relevant studies, which entails assessing the recall of the search strategy. Additionally, technologies that assist in automating or semi-automating steps of the evidence synthesis process, especially those related to identifying relevant evidence, have been proposed to help address the well-known challenges in the evidence synthesis process [5].

In the field of temporomandibular disorders (TMDs), which corresponds to a group of musculoskeletal and neuromuscular conditions that affect the temporomandibular joints (TMJ), masticatory muscles and their associated structures [6], the literature has consistently highlighted complexities due to its definition and scope. There is a clear challenge in categorizing these conditions collectively covered under the umbrella term “temporomandibular disorders” [7]. Several terminologies and taxonomies are used in contemporary TMD scientific research, hindering the development of highly sensitive SS for identifying reliable evidence related to these conditions. Identifying and synthesizing the evidence for TMDs is essential for accurate diagnosis, treatment planning, and improved patient outcomes while avoiding unnecessary treatments, promoting cost-effectiveness, and standardizing clinical practice. Thus, understanding the available evidence for TMDs is critical to optimize patient care and outcomes [8].

Systematically identifying all relevant and specific terms is crucial in the development of a comprehensive and highly sensitive SS and an automated classifier in the field of TMDs. This approach is essential for retrieving comprehensive information on this topic, ensuring that valuable insights are not inadvertently omitted due to reliance on particular terminology. Furthermore, the validation of this strategy plays a crucial role in refining and optimizing the search process, enhancing its accuracy and reliability, and ensuring that all relevant data are captured effectively. This study aimed to develop and validate a search strategy and an automated classifier for identifying primary studies in the TMD field.

## 2. Methods

We developed a Boolean SS and an AC for identifying TMD studies using an iterative process: (1) Initial development; (2) Test of the performance and refinement; (3) Final validation; and (4) Audit.

### 2.1 Initial development

The creation of a first version of the SS and the AC required two stages, briefly summarized as:

#### 2.1.1 TMD category definition

For this study, we set a definition of “TMD-relevant research evidence” based on the most widely used guidelines and classifications for these conditions, including the American Academy of Orofacial Pain [6], International Classification for Orofacial Pain [9], Diagnostic Criteria for Temporomandibular Disorders (DC/TMD) [10] and the Expanded Classification of TMD from Peck* et al*. [7].

#### 2.1.2 Identification and selection of TMD-relevant terms

To devise a comprehensive list of relevant terms for the Boolean SS and AC development, we manually reviewed the search terms reported in the search strategies of 71 TMD-related SRs published from 2019 to 2021 available in the Epistemonikos database [11]. Other relevant terms were obtained by applying Word2vec technology to the corpus of documents available in the repository [12] with the proprietary software of Epistemonikos. The TMD-relevant search terms were combined using Boolean operators OR/AND to create the first version of the Boolean SS.

On the other hand, the list of terms for the binary exact-match classifiers was obtained by applying Word2vec technology [12]. The terms with the more similar vectors were analyzed by a team of content and method experts and selected based on their incremental recall (*i.e.*, the capacity to identify new “positives” in the unclassified records). The classifier votes as “yes” to the records with an exact match with at least one of the terms. The algorithm is revised and improved iteratively based on discrepancies with classification by human users.

### 2.2 Test of the performance and refinement

The performance of the Boolean SS and the AC for retrieving TMD studies was assessed by the relative recall method. The relative recall is the proportion of articles that a specific search or/and automatic classifier retrieves of the total relevant studies determined by a reference standard [13]. Our initial reference standard was composed of all the primary studies included in a random sample of 100 TMD-relevant SRs published from 2000 to 2022, identified through the Epistemonikos database. The eligibility criteria applied to the TMD-relevant SRs were:

(a) Fulfilled the definition of systematic review used in the Epistemonikos Database [2].

(b) Provided a clear description of the list of the included primary studies.

(c) Addressed a question directly related to TMD. Reviews focusing on a broader topic (*e.g.*, chronic pain or dentistry), or TMD and other conditions (*e.g.*, other orofacial diseases) were excluded.

To evaluate the relative recall of the SS and the AC, the 100 selected TMD-relevant SRs were randomly divided into five sets of 20 SRs each. The relative recall of the Boolean SS and the AC was assessed separately against each group of primary studies included in 20 SRs using an iterative process. To improve the designed Boolean SS and the AC, we examined the terms used by the primary studies that were not retrieved by the SS and/or the AC, and we added them accordingly. After each set, additional terms (*e.g.*, cervico-craniofacial, oromandibular, re-ankylosis) along with modifications to the search syntax (*e.g.*, add parenthesis to group terms, or use asterisks as a wildcard to truncate terms) enhanced the SS and fed the AC to improve its performance.

We retested its performance against the new set of primary studies included in 20-SRs five times and similarly to those used in the previous step (Fig. 1). Only TMD-relevant primary studies with an abstract were included in the analysis.

**Fig. 1.** Illustration of the sequential steps for search strategy and automated classifier performance analysis and improvement. TMD: Temporomandibular disorders; SRs: Systematic reviews; SS: Search strategy; AC: Automated classifier. In each set, we evaluated the SS and the AC performance against all primary studies included in 20 TMD-relevant SRs. After each set, we added new terms and modified the search syntax to improve its performance.

In each set, the data extracted from the selected SRs were title, authors, year of publication, type of question (prevention or treatment, etiology, epidemiology, diagnosis, and prognosis), and the number of included primary studies. From the primary studies, title, year of publication, and study design (Randomized controlled trial or Study designs different from randomized controlled trial) were extracted.

Comprehensiveness was calculated (sensitivity or relative recall) of the Boolean SS and the AC classifier as:

$$\frac{Number\;of\;studies\;retrieved\;by\;the\;SS/AC\;}{\text{Total references in the reference standard}}\;\times 100$$

### 2.3 Validation

In order to test the generalizability of the final version of the TMD SS and AC, we calculate the performance against all the primary studies included in TMD-relevant SRs published from January to April 2023.

### 2.4 Audit

To understand the reasons for the failure in the identification of articles, we conducted an audit of all references that were not retrieved by the SS or were not classified as TMD-relevant by the AC (*e.g.*, machine error, missing term, reference format problem,* etc.*). These studies were not added to the numerator of the comprehensiveness calculation.

All the processes mentioned above were incorporated into the online LOVE platform for TMD hosted at https://app.iloveevidence.com/tmd.

## 3. Results

### 3.1 Initial development

The TMD standard utilized for the algorithm creation was defined as “Any article related to temporomandibular disorders (TMD) (including orofacial pain with a musculoskeletal origin or any alteration of the TMJ). We include articles assessing any question (*i.e.*, treatment, diagnostics, etiology, epidemiology, prognosis). Articles about other pathologies that are related to TMD are also included. We exclude articles related to other causes of orofacial pain, such as neuropathic pain, neurovascular pain, idiopathic pain, or other causes of pain in the facial area”.

### 3.2 Identification and selection of TMD-relevant terms

A total of 61 different specific terms related to TMD were identified by reviewing the search terms used in the strategies of 71 TMD-related SRs published from 2019 to 2021. The analysis excluded non-specific TMD terms, such as orofacial pain, myofascial pain syndrome, craniofacial pain, and others. The most utilized terms in the 71 TMD-related SRs was “temporomandibular disorders”, mentioned in 27 of them (38%), followed by the term “temporomandibular joint disorders”, present in 26 (37%), and “temporomandibular joint”, present in 26 (37%). **Supplementary Table 1** presents the first Boolean SS and the terms selected for the AC classifier.

The analysis excluded terms such as “orofacial pain”, “myofascial pain syndrome”, and “craniofacial pain” because they lacked specificity to the TMD term.

### 3.3 Test of the performance and refinement

A total of 100 random SRs published from 2002–2022 were included for analysis. The research question domain of the SRs was: 65 SRs were about prevention and treatment (65%), 7 about etiology (7%), 7 about epidemiology (7%), 7 about diagnosis (7%), 2 about prognosis (2%), and 12 SRs (12%) addressed more than one research question. Thirty-seven of the selected SRs included only randomized controlled trials (RCTs) (37%), and the range of included primary studies per SR varied from 2 to 113. A total of 1297 primary studies were included in the 100 selected SRs, but 26 of them were excluded from the analysis (n = 7 not considered as a TMD-related article, n = 19 no abstract). The description of each set is presented in Table 1. The SS of each set is available in the **Supplementary material**.

**Table 1.** Description of sets for search strategy and automated classifier performance and refinement.

Set number (N SRs)	Range of publication year of the SRs	SR research question domain (n; %)	Sum of Primary Studies Included (n)	Primary Studies Included per SR (min–max)	SR of RCTs only (n; %)	Excluded Primary Studies (n; Non-TMD = n, wo/abstract = n)	Final number of Primary Studies
Set 1 (20 SRs)	2002–2020	Prevention and Treatment 14; 70% Etiology 2; 10% Epidemiology 2; 10% Diagnosis 1; 5% More than one question domain 1; 5%	205	3–45	9; 45%	9; Non-TMD = 4, wo/abstract = 5	196
Set 2 (20 SRs)	2006–2020	Prevention and Treatment 13; 65% Epidemiology 2; 10% Prognosis 1; 5% Etiology 1; 5% Diagnosis 1; 5% More than one Question 2; 10%	252	4–36	7; 35%	4; Non-TMD = 1, wo/abstract = 3	248
Set 3 (20 SRs)	2007–2021	Prevention and Treatment 12; 60% Diagnosis 2; 10% Etiology 1; 5% More than one Question 5; 25%	280	2–43	5; 25%	3; Non-TMD = 2, wo/abstract = 1	277
Set 4 (20 SRs)	2009–2022	Prevention and Treatment 11; 55% Diagnosis 3; 15% Etiology 1; 5% Prognosis 1; 5% More than one Question 4; 20%	311	3–113	6; 30%	6; Non-TMD = 0, wo/abstract = 6	305
Set 5 (20 SRs)	2006–2021	Prevention and Treatment 15; 75% Epidemiology 3; 15% Etiology 2; 10%	223	3–31	10; 50%	4; Non-TMD = 0, wo/abstract = 4	219

*Table 1 provides a description of TMD-SR recall and details the success rate of the method. Those findings guided modifications made to subsequent iterations and sets. The six different sets that were performed are outlined. Min: minimum; max: maximum; N or n: number/quantity; RCTS: Randomized Controlled Trials; SR: Systematic Reviews; TMD: Temporomandibular Disorders, wo: without.*

In the first set of references, a sensitivity of 89.34% and 70.05% was obtained for the SS and AC respectively. This means that the SS detected 175 primary studies, while the TMD AC detected 137 out of 196 primary studies. After the 5th set, the SS achieved a recall of 99.54% (yield 218 out of 219 primary studies), while a recall of 89.5% was obtained by the AC studies (yield 196 out of 219 primary studies). This represents an improvement of 10.2% for SS sensitivity and 19.45% for the AC classifier. The relative recall result for each set is presented in Table 2.

**Table 2.** Relative recall results per set of references.

	Relative recall			
	TMD Boolean search strategy		TMD-automated classifier	
	rPS/tPS	%	rPS/tPS	%
Set 1	175/196	89.34%	137/196	70.05%
Set 2	233/248	93.95%	210/248	84.68%
Set 3	264/277	95.31%	230/277	83.03%
Set 4	292/305	95.74%	282/305	92.46%
Set 5	218/219	99.54%	196/219	89.50%

*Each set is composed of 20 TMD-relevant SRs. rPS: number of retrieved primary studies; tPS: total number of primary studies included in the set; TMD: temporomandibular disorders.*

### 3.4 Validation

A total of 28 SRs published between January and April 2023 were included for the final validation. The included SRs answered questions about prevention and/or treatment (n = 15, 53.57%), prognosis (n = 7, 25%), diagnosis (n = 3, 10.71%), epidemiology (n = 2, 7.14%), and etiology (n = 1, 3.57%). The number of primary studies included in the SRs ranged from 3 to 43. Twelve SRs (42.86%) included only RCTs in their analysis, while 57.14% included other primary study designs. In total, 307 primary studies were included in the 28 SRs, but five of them were excluded from the analysis (n = 4 not considered as a TMD-related article, n = 1 no abstract). Finally, 301 primary studies were analyzed. The sensitivity of the search strategy was 99.67%, yielding 300 out of 301 primary studies, and the sensitivity of the TMD AC detected 88.04% of the studies (265 of 301).

### 3.5 Audit results

All references retrieved by the final SS were fully analyzed. Only one study was not retrieved as TMD-relevant due to the use of a specific term not included in the previous SS (“MTrPs”; Myofascial trigger points in the masseter muscle). The AC did not retrieve 36 studies (11.96%). Fifteen of them (41.66%) include a new group of terms not included previously in the AC (*e.g.*, “Temporal Mandibular Joint Disorders”, “temporomandibular joint disc perforation”, “sub-condylar mandibular fractures”), while the other 21 articles (58.33%) did not present TMD-specific terms (*e.g.*, “latent myofascial trigger points”, “surgical management of subcondylar fractures”, “myofascial pain”, *etc.*). Table 3 presents the final Boolean SS. The final list of relevant terms for the binary exact-match classifiers is available in the **Supplementary Table 2**.

**Table 3.** Final Boolean search strategy terms for temporomandibular disorders studies.

Final version of the search strategy
((temporomandibular* OR “temporo-mandibular” OR craniomandibular* OR “cranio-mandibular” OR “cranio-cervical-mandibular” OR “cervico-craniofacial” OR tmj OR oromandibular*) AND (arthralgi* OR disorder* OR disease* OR dysfunct* OR disfunct* OR pain* OR noise* OR sound* OR arthrocentesis* OR condylectomy* OR degenerativ* OR subluxation* OR osteoarthrit* OR arthrit* OR ankylosis* OR “re-ankylosis” OR reankylosis* OR arthrosis* OR hypermobilit* OR involvement* OR displac* OR problem* OR inflammat* OR surger* OR symptom* OR sign* OR dislocation* OR fracture* OR effusion* OR derangement* OR arthropat* OR lock*)) OR ((masticator* OR masseter* OR temporal* OR pterygoid* OR orofacial*) AND (pain* OR myalgi* OR atroph* OR hypertroph* OR headache* OR dystoni* OR dysfunct* OR disfunct* OR myogenous* OR myofascial*)) OR tmd OR ddwr OR “tmd-related headache” OR ddwor OR tmds OR tmjd OR “mandibular dysfunction” OR helkimo OR wilkes* OR rdctmd OR “facial myalgia” OR “disc displacement without reduction” OR ((condylar* OR subcondylar* OR “mandibular condyle” OR “mandibular condyles”) AND (hyperplasia* OR resorption* OR fracture* OR degenerat*))

*: Symbol use for wildcard searching which, when included at the end or within a word, instructs the database to search for all forms of it.

## 4. Discussion

To the best of our knowledge, this study developed the first validated SS for TMD research articles with excellent performance. An AC was also created, supported by a free platform and capable of automatically and efficiently identifying published TMD-related research articles. This allows users to have, in one place, including relevant electronic databases, the available TMD systematic reviews and primary studies from the full spectrum of disorders.

In the process of designing the TMD strategy and algorithm, it was initially necessary to identify the terminology employed in the literature to denote Temporomandibular Disorders. A wide variety of general terms related to Temporomandibular Disorders (TMD) and non-specific terms pertaining to TMD have been employed in publications. This phenomenon can be attributed primarily to the utilization of specialized acronyms, which are not widely recognized or understood outside of specific domains. Examples of inconvenient terms are “tmjid” for temporomandibular joint internal derangement, “tmj ccl” for temporomandibular joint chronic close lock and “cmd” for craniomandibular disorders. The only outcome for these acronyms was the article that uses them [14, 15]. Additionally, the inclusion of terms that are important only within a specific global context represented a difficulty for inclusion (*e.g.*, “anteriorly dislocated disks”; “disk recapturing bite plate”). Those terms are not specific to TMD but, in a particular context, may have a value and therefore contribute to identifying specific research articles. Authors are then suggested to utilize high-impact influential terms to ease evidence capture and further mapping.

An iterative process is crucial to improve the sensitivity of an SS. When search engines present decreased sensitivity, articles are not retrieved after search queries or by using an algorithm. When searches are performed in electronic databases, search engines use the information available in title and abstract. Unfortunately, research articles lacking an abstract are difficult to retrieve. Acknowledging the limitations of SS or any classifier using a language-based technique to manage records without an abstract, we decided to exclude references without an abstract [2].

A similar methodological study validating a search strategy for randomized clinical trials related to periodontitis was published, achieving a sensitivity of 93.2% [16]. In this study, the SS was evaluated against a gold standard composed of 55 RCTs related to periodontitis. In contrast, in our study the reference standard was composed of more than 100 primary studies manually checked and included in a random sample of 100 TMD-relevant SRs published from 2000 to 2022. That study also calculated specificity, precision, and number needed to read, values that were not measured in this TMD project [16]. The calculation of specificity was not performed for the LOVE TMD platform due to the prioritization of achieving a high sensitivity as the primary goal. Subsequently, and in a later phase, adjustments should be made to enhance the specificity for the categorization of articles based on taxonomic trees. There are other examples in the literature of methodological studies that aim to develop and validate a highly sensitive search strategy for retrieving studies by using the relative recall method, such as limb prostheses and patients’ views and preferences [17, 18].

In subsequent iterations, enhancements can be made to the LOVE TMD platform algorithm in order to attain outcomes with greater sensitivity. Although the algorithm in question shows valuable sensitivity, it is interesting to note that, on average, artificial intelligence (AI) algorithms typically exhibit an increasing sensitivity when instructed. While other platform algorithms are enhanced to user experience [18], are partially known [19] or depend on the query made [20], this algorithm works with better and complete recall. Thus, the utilization of an electronic platform for hosting all the relevant research sources on TMD has the advantage of time efficiency by eliminating the need for manual application of search criteria and subsequent selection [21]. This feature enables the future application of enhanced usability to the platform. For instance, it facilitates the categorization of reviews based on their corresponding research questions. Consequently, this categorization may be utilized to generate automatically fed taxonomic trees, which in turn will promote evidence-based decision-making. As recommended by other organizations like the US National Academy of Medicine [8], it is advisable to create evidence-decision-making, where maps of present science will be crucial. These maps will provide and identify areas where information is lacking, thereby allowing the development of novel concepts for human initiatives in both basic and clinical sciences.

Considering that the SS was tested only in the Epistemonikos database may represent a limitation of this study, since it restricts the extrapolation of the performance of the SS developed to other databases. Nevertheless, adapting search syntax to other electronic databases is a common procedure for information specialists and researchers with expertise in evidence synthesis. Additionally, the proposed SS was validated against a randomly selected and heterogeneous gold standard of 1271 articles from different study designs that answer various question domains, minimizing the potential problem of generalization.

Finally, this trained and validated AC may ultimately enhance the evidence synthesis streamline and evidence-based decision-making within the field of TMD research. Even though AC has demonstrated promising results, there is still an important risk of missing relevant information by using automated methods. Human checking of data is still therefore needed.

## 5. Conclusions

The proposed SS demonstrates excellent performance for retrieving research articles related to TMD. Additionally, the AC will support an open access and constantly updated digital repository to gather all the relevant evidence about TMD with good sensitivity.

## Supplementary Material


