# Supplementary material for: Development and validation of a search strategy and an automated classifier for retrieving temporomandibular disorders studies
Source: J Oral Facial Pain Headache. 2024 Jun 12;38(2):74–81. doi: 10.22514/jofph.2024.015 (PMC11810670; doi:10.22514/jofph.2024.015)
Supplement: Supplementary file 1 [file Supplementary-material.docx]

Supplementary material

Supplementary Table 1. Search strategies used in each set.

| Search strategy used in set 1 | (((temporomandibular* OR craniomandibular* OR tmj) AND (arthralgia* OR disorder* OR dysfunct* OR pain* OR osteoarthrit* OR ​​sound* OR noise* OR arthrocentesis* OR condylectomy* OR degenerative OR subluxation OR arthrit*)) OR ((“masticatory muscles” OR “masticatory muscle” OR “masseter muscle” OR “temporal muscle” OR "masseter muscles” OR “temporal muscles) AND (pain* OR mialgia* OR atrophy)) OR (tmd OR ddwr OR “tmd-related headache” OR ddwor OR tmds) OR “disc displacement” OR “disc disorders” OR “orofacial pain” OR “orofacial dystonia” OR “temporomandibular joint surgery” OR (condylar AND (hyperplasia* OR resorption*))) |
| --- | --- |
| Search strategy used in set 2 | (((temporomandibular* OR “temporo-mandibular” OR craniomandibular* OR “cranio-mandibular” OR “cranio-cervical-mandibular” OR tmj) AND (arthralgia* OR disorder* OR disease* OR dysfunct* OR disfunct* OR pain* OR ​​sound* OR noise* OR arthrocentesis* OR condylectomy* OR degenerative OR subluxation OR osteoarthrit* OR arthrit* OR ankylosis* OR “internal derangement” OR “re-ankylosis” OR “reankylosis” OR hypermobilit* OR involvement* OR displacement OR problem* OR inflammation* OR surger* OR symptom* OR signs*)) OR ((masticator* OR masseter* OR temporal* OR myogenous* OR myofascial* OR orofacial*) AND (pain* OR myalgi* OR atroph* OR hipertroph* OR headache* OR dystoni*)) OR (tmd OR ddwr OR “tmd-related headache” OR ddwor OR tmds OR “mandibular dysfunction” OR helkimo OR wilkes* OR rdctmd OR (condylar AND (hyperplasia* OR resorption*)))) |
| Search strategy used in set 3 | (((temporomandibular* OR “temporo-mandibular” OR craniomandibular* OR “cranio-mandibular” OR “cranio-cervical-mandibular” OR tmj) AND (arthralgia* OR disorder* OR disease* OR dysfunct* OR disfunct* OR pain* OR ​​sound* OR noise* OR arthrocentesis* OR condylectomy* OR degenerative OR subluxation OR osteoarthrit* OR arthrit* OR ankylosis* OR "internal derangement”OR “re-ankylosis” OR “reankylosis” OR hypermobilit* OR involvement* OR displacement OR problem* OR inflammation* OR surger* OR symptom* OR signs* OR dislocation* OR fracture*)) OR ((masticator* OR masseter* OR temporal* OR pterygoid* OR myogenous* OR myofascial* OR orofacial*) AND (pain* OR myalgi* OR atroph* OR hipertroph* OR headache* OR dystoni* OR dysfunct* OR disfunct*)) OR (tmd OR ddwr OR “tmd-related headache” OR ddwor OR tmds OR tmjd OR “mandibular dysfunction” OR helkimo OR wilkes* OR rdctmd OR (condylar AND (hyperplasia* OR resorption* OR fracture*)))) |
| Search strategy used in set 4 | (((temporomandibular* OR “temporo-mandibular” OR craniomandibular* OR “cranio-mandibular” OR “cranio-cervical-mandibular” OR “cervico-craniofacial” OR tmj OR oromandibular*) AND (arthralgia* OR disorder* OR disease* OR dysfunct* OR disfunct* OR pain* OR ​​sound* OR noise* OR arthrocentesis* OR condylectomy* OR degenerative OR subluxation OR osteoarthrit* OR arthrit* OR ankylosis* OR “re-ankylosis” OR “reankylosis” OR hypermobilit* OR involvement* OR displacement OR problem* OR inflammation* OR surger* OR symptom* OR signs* OR dislocation* OR fracture* OR effusion OR derangement* OR arthropathy)) OR ((masticator* OR masseter* OR temporal* OR pterygoid* OR myogenous* OR myofascial* OR orofacial*) AND (pain* OR myalgi* OR atroph* OR hipertroph* OR headache* OR dystoni* OR dysfunct* OR disfunct*)) OR (tmd OR ddwr OR “tmd-related headache” OR ddwor OR tmds OR tmjd OR “mandibular dysfunction” OR helkimo OR wilkes* OR rdctmd OR ((condylar* OR subcondylar* OR “mandibular condyle” OR “mandibular condyles”) AND (hyperplasia* OR resorption* OR fracture* OR degenerat*)))) |
| Search strategy used in set 5 | (((temporomandibular* OR “temporo-mandibular” OR craniomandibular* OR “cranio-mandibular”OR “cranio-cervical-mandibular” OR “cervico-craniofacial”OR tmj OR oromandibular*) AND (arthralgi* OR disorder* OR disease* OR dysfunct* OR disfunct* OR pain* OR ​​sound* OR noise* OR arthrocentesis* OR condylectomy* OR degenerativ* OR subluxation* OR osteoarthrit* OR arthrit* OR ankylosis* OR “re-ankylosis” OR reankylosis* OR arthrosis* OR hypermobilit* OR involvement* OR displac* OR problem* OR inflammat* OR surger* OR symptom* OR sign* OR dislocation* OR fracture* OR effusion* OR derangement* OR arthropat* OR lock*)) OR ((masticator* OR masseter* OR temporal* OR pterygoid* OR myogenous* OR myofascial* OR orofacial*) AND (pain* OR myalgi* OR atroph* OR hipertroph* OR headache* OR dystoni* OR dysfunct* OR disfunct*)) OR tmd OR ddwr OR “tmd-related headache” OR ddwor OR tmds OR tmjd OR “mandibular dysfunction”OR helkimo OR wilkes* OR rdctmd OR “facial myalgia” OR “disc displacement without reduction”OR ((condylar* OR subcondylar* OR “mandibular condyle”OR “mandibular condyles”) AND (hyperplasia* OR resorption* OR fracture* OR degenerat*))) |

Supplementary Table 2. Final list of relevant terms for the binary exact-match classifiers.

| temporomandibular disorder  temporomandibular joint disorders  craniomandibular disorders  temporomandibular dysfunction  tmds  masticatory muscle pain  tmj dysfunction  temporomandibular joint dysfunction  tmj disorders  temporomandibular joint disorder  tmd patients  anterior disc displacement  masticatory myofascial pain  temporomandibular joint pain  temporomandibular pain  temporomandibular joint dysfunction syndrome  temporomandibular disorder pain  orofacial dystonia  temporomandibular joint osteoarthritis  ​​tmj dysfunction  tmd symptoms  temporomandibular disorders  disc displacement  condylar hyperplasia  disc displacement without reduction  temporomandibular joint  craniomandibular dysfunction  muscular tmd  temporomandibular joint sounds  idiopathic condylar resorption  ddwr  tmd-related headache  ddwor  tmj noise  symptoms of tmj ddr  temporomandibular joint derangements  temporomandibular joint disc displacement  tmj ddr  tmj disc displacement  dextrose prolotherapy in temporomandibular joint hypermobility treatment  assess tmj inflammation  adult patients with bilateral temporomandibular joint hypermobility  growth factors after temporomandibular joint arthroscopy  rdctmd ia  temporomandibular joint ankylosis  management of temporomandibular joint ankylosis  outcome of temporomandibular joint internal derangement  concomitant tmj surgery  symptomatic tmj hypermobility  efficacy of temporomandibular joint ankylosis  clinical evaluation in tmid patients  early release of tmj ankylosis  effects of myogenous facial pain  active tmj arthritis  indications for temporomandibular joint reconstruction  surgical treatment of temporomandibular joint ankylosis  manage temporomandibular joint ankylosis  increased mmp-13 immunoreactivity in tmj  active bilateral idiopathic condylar resorption  acute tmj arthritis  symptoms of tmj mp  apparent post-operative condylar resorption  temporomandibular joint ankyloses  myogenous temporomandibular disorder patients  injections into masticatory muscles  patients with temporomandibular joint ankylosis  craniomandibular reankylosis  chronic myogenous orofacial pain  masseter muscle pain  painful tmj area  condylar resorption  cranio-cervical-mandibular dysfunction  etiology of temporomandibular joint dysfunction-pain syndrome  acute temporomandibular muscle pain dysfunction  post-occlusal splint treatment of temporomandibular joint disturbance syndrome  myofascial pain-dysfunction temporomandibular patients  tmjmuscle pain dysfunction  assessment of mandibular dysfunction  clinical dysfunction indices of helkimo  etiology of mandibular dysfunction  initial assessment of tmj inflammation  bilateral temporomandibular joint hypermobility  patients with wilkes stage iv  rdctmd ib  temporomandibular joint ankylosis in children  management of tmj  temporomandibular joint internal derangement  temporomandibular joint hypermobility  temporo-mandibular joint intracapsular disease  managing tmj ankylosis  myogenous facial pain  early diagnosis of temporomandibular joint involvement  temporomandibular joint reconstruction  involvement in tmj disc histopathology  active bilateral idiopathic condylar resorption confirmed  myofascial pain in bruxers  chronic changes of tmj arthritis  temporomandibular joint myofascial pain  temporomandibular joint ankylosis causes limitation  tmj ankylosis  temporomandibular disorder patients  management of tmj re-ankylosis  jaw muscle pain  masseter muscle pain evoked  developed progressive condylar resorption following  condylar resorption after orthognathic surgery  craniomandibular disfunction treatment  cranio-mandibular disorder  temporomandibular joint dysfunction-pain syndrome  temporomandibular joint disturbance syndrome  pain-dysfunction temporomandibular patients  treatment of tmjmuscle pain dysfunction  mandibular dysfunction  incidence of mandibular dysfunction  significant improvement in tmj pain  temporomandibular joint involvement  bilateral temporomandibular joint hypermobility referred  temporomandibular joint arthroscopy  patients of tmj ankylosis  tmj internal derangement  management of condylar resorption  tmj hypermobility  patients experiencing myogenous facial pain compared  temporomandibular joint ankylosis from  tmj tumor defects  temporomandibular joint ankylosis poses  tmj disc histopathology  active bilateral idiopathic condylar resorption treated  myofascial pain symptoms in bruxers  chronic tmj disease  temporomandibular joint myofascial pain  treatment of temporomandibular joint ankylosis  treating tmj ankylosis  temporomandibular joint ankylosis treated  modified temporalis anchorage in craniomandibular reankylosis  jaw muscle pain in females  masseter muscles in myofascial pain patients  progressive condylar resorption  myogenic cranio-cervical-mandibular dysfunction  development of condylar resorption  craniomandibular disorder  temporomandibular muscle pain dysfunction  tmjds  temporomandibular patients  temporomandibular joint involvement in childhood arthritis  outcome variables of tmj hypermobility  temporomandibular joint arthroscopy improve outcomes  postoperative jaw opening exercises  treatment of temporomandibular joint internal derangement  post surgical tmj medical management  tmj pain  tmid patients  tmj ankylosis in delta nile  patients with myogenous facial pain  tmj internal derangement suggests  active idiopathic condylar resorption  detection of tmj disease  tmd clinic  patients with preoperative condylar resorption  treatment of tmj ankylosis  tmj re-ankylosis  severe jaw muscle pain  reducing masseter muscle pain  progressive condylar resorption following orthognathic surgery  subject of condylar resorption  patients with craniomandibular disorder  craniomandibular disorder with respect  treat acute temporomandibular muscle pain dysfunction  temporomandibular joint hypermobility treatment  tmj capsular width  wilkes stage iv  wilkes stage  study selection included preexisting cbct condylar resorption  tmj pain on palpation  temporomandibular joint ankylosis surgical treatment  tmid patients maximum masseter activation  treatment modalities of tmj ankylosis  active idiopathic condylar resorption treated  high prevalence of temporomandibular joint arthritis  tmj mp  post-operative condylar resorption  patients with myogenic cranio-cervical-mandibular dysfunction  temporo-mandibular-joint complaints  tmj inflammation  temporomandibular joint hypermobility?  wilkes stage iv internal derangement  surgical tmj  treatment of symptomatic tmj hypermobility  various types of tmj ankylosis  bilateral condylectomy  treatment of temporomandibular joint myofascial pain  preoperative condylar resorption  tolerable temporo-mandibular-joint complaints  tmj hypermobility treatment  wilkes stage iv internal derangement?  tmj ankylosis seeking treatment  wilkes stage iv from  treatment of temporomandibular joint hypermobility  treatment of temporomandibular joint myofascial pain  myogenous cranio-mandibular disorder  tmj locking  tmj hypermobility assessed  wilkes stage iv presenting  verbal scale expressing tmj pain  patients underwent bilateral condylectomy  prevalence of temporomandibular joint disease  simple type of myogenous cranio-mandibular disorder  tmj pain intensity  tmj prolotherapy  wilkes stages  wilkes stages ii  wilkes stages ii through v  signs of tmj involvement  tmj arthritis  tmj involvement  patients with active idiopathic condylar resorption  temporomandibular joint arthritis  temporomandibular joint disease  tmj disease  research diagnostic criteria for temporomandibular disorders diagnosis  rdctmd  temporomandibular joint internal derangements  treatment of temporomandibular joint internal derangements  chronic tmj degenerative disorders  management of chronic tmj degenerative disorders  temporomandibular joint degenerative disorders  temporomandibular disorder cases  temporomandibular joint internal derangements assessed  jaw muscle pain reduction  clinical pain among myofascial temporomandibular disorder participants  cohort of myofascial temporomandibular disorder participants  myofascial temporomandibular disorder participants  temporomandibular disorder participants  chronic tmjd pain  clinically significant tmjd pain  progression of chronic tmjd pain  significant tmjd pain  tmjd  tmjd chronicity  tmjd pain  tmjd patients  tmjd patients develop severe disorders associated  chronic recurrent temporomandibular joint dislocation  chronic recurrent tmj dislocation  patients with chronic recurrent tmj dislocation  recurrent tmj dislocation  temporomandibular joint dislocation tmj for treatment  treatment of chronic recurrent tmj dislocation  recurrent temporomandibular joint dislocation  temporomandibular joint dislocation  treatments for recurrent temporomandibular joint dislocation  mandibular condylar fractures  temporomandibular joints with closed treatment  treatment of mandibular condylar fractures  chronic tmd pain  patients with tmd pain  temporomandibular dysfunction pain  mandibular subcondylar fractures  maxillomandibular fixation for subcondylar fractures  treatment of mandibular subcondylar fractures  anterior displaced temporo anterior displaced temporomandibular discs | temporomandibular joint arthrocentesis for acute  tmj arthrocentesis  tmj procedures  nonreducing temporomandibular joint disk displacement  temporomandibular joint arthrography  temporomandibular joint arthrography alone  patients with temporomandibular joint osteoarthrosis  temporomandibular joint osteoarthrosis  tmj osteoarthrosis  classification of temporomandibular joint osteoarthrosis  temporomandibular joint syndrome  tmj for treatment  anterior displaced temporomandibular discs  temporomandibular joint effusion  tmj effusion  subcondylar fractures of the mandible  arthropathy of the temporomandibular joint in children  cervico-craniofacial pain  cervico-craniofacial pain of myofascial origin  chronic masticatory muscle pain patients  chronic masticatory muscle pain patients for psychosocial functioning  masticatory muscle pain patients  temporomandibular disorder signs  temporomandibular signs  tmd  tmd signs  disk displacement with reduction  temporomandibular disk displacement  temporomandibular disk displacement with reduction  temporomandibular joint clicking  tmj clicking  bilateral temporomandibular joint symptomatology  presence of significant temporomandibular joint pathology  significant temporomandibular joint pathology  temporomandibular joint pathology  temporomandibular joint symptomatology  temporomandibular disorders patients  temporomandibular disorders patients present  tmj intracapsular pain  tmj intracapsular pain according  temporomandibular joint symptoms  temporomandibular disorder pain dysfunction syndrome  temporomandibular disorder pain dysfunction syndrome affect dietary intake?  arthritic mandibular condyles  post-traumatic tmd  trauma-induced tmd  temporomandibular joint pain-dysfunction syndrome  temporomandibular joints with reciprocal clicking  treatment of temporomandibular joints  fracture of the mandibular condyles  chronic facial myalgia  patients facial myalgia  facial myalgia patients  myalgia treatment of masticatory muscles  unilateral tmj internal derangement  temporomandibular joint problem  masticatory myofascial pain disorders  treatment of masticatory myofascial pain disorders  myofascial face pain  patients with myofascial face pain  treating myofascial face pain  women with myofascial face pain  tmj internal derangements  closed jaw locking  temporomandibular joint arthrosis  tmj arthrosis  tmj arthrosis with closed lock  treating tmj arthrosis  disk displacement without reduction  temporomandibular joint locking  treatment of temporomandibular joint locking  chronic closed lock  temporomandibular joint disc repositioning  tmj articular disc repositioning  tmj disc repositioning  trigger points in masticatory muscles  temporomandibular myofascial pain  treatment of temporomandibular myofascial pain  craniofacial myalgia  patients with craniofacial myalgia  painful temporomandibular joints  treatment of painful temporomandibular joints  temporomandibular joint interal derangement  tmjid  displaced temporomandibular joint meniscus  temporomandibular joints with anterior meniscus displacement  temporomandibular disorder  temporomandibular joint disorders  craniomandibular disorders  temporomandibular dysfunction  tmds  masticatory muscle pain  tmj dysfunction  temporomandibular joint dysfunction  tmj disorders  temporomandibular joint disorder  anterior disc displacement  masticatory myofascial pain  temporomandibular joint pain  temporomandibular pain  temporomandibular joint dysfunction syndrome  temporomandibular disorder pain  orofacial dystonia  temporomandibular joint osteoarthritis  ​​tmj dysfunction  tmd symptoms  temporomandibular disorders  disc displacement  condylar hyperplasia  disc displacement without reduction  temporomandibular joint  craniomandibular dysfunction  muscular tmd  temporomandibular joint sounds  idiopathic condylar resorption  ddwr  tmd-related headache  ddwor  tmj noise  symptoms of tmj ddr  temporomandibular joint derangements  temporomandibular joint disc displacement  tmj ddr  tmj disc displacement  dextrose prolotherapy in temporomandibular joint hypermobility treatment  assess tmj inflammation  growth factors after temporomandibular joint arthroscopy  rdctmd ia  temporomandibular joint ankylosis  management of temporomandibular joint ankylosis  outcome of temporomandibular joint internal derangement  concomitant tmj surgery  symptomatic tmj hypermobility  efficacy of temporomandibular joint ankylosis  early release of tmj ankylosis  effects of myogenous facial pain  active tmj arthritis  indications for temporomandibular joint reconstruction  surgical treatment of temporomandibular joint ankylosis  manage temporomandibular joint ankylosis  increased mmp-13 immunoreactivity in tmj  active bilateral idiopathic condylar resorption  acute tmj arthritis  symptoms of tmj mp  apparent post-operative condylar resorption  injections into masticatory muscles  chronic myogenous orofacial pain  masseter muscle pain  painful tmj area  condylar resorption  cranio-cervical-mandibular dysfunction  etiology of temporomandibular joint dysfunction-pain syndrome  acute temporomandibular muscle pain dysfunction  post-occlusal splint treatment of temporomandibular joint disturbance syndrome  tmjmuscle pain dysfunction  assessment of mandibular dysfunction  clinical dysfunction indices of helkimo  etiology of mandibular dysfunction  initial assessment of tmj inflammation  bilateral temporomandibular joint hypermobility  rdctmd ib  management of tmj  temporomandibular joint internal derangement  temporomandibular joint hypermobility  temporo-mandibular joint intracapsular disease  managing tmj ankylosis  myogenous facial pain  early diagnosis of temporomandibular joint involvement  temporomandibular joint reconstruction  involvement in tmj disc histopathology  active bilateral idiopathic condylar resorption confirmed  myofascial pain in bruxers  chronic changes of tmj arthritis  temporomandibular joint myofascial pain  temporomandibular joint ankylosis causes limitation  tmj ankylosis  management of tmj re-ankylosis  jaw muscle pain  masseter muscle pain evoked  developed progressive condylar resorption following  condylar resorption after orthognathic surgery  craniomandibular disfunction treatment  cranio-mandibular disorder  temporomandibular joint dysfunction-pain syndrome  temporomandibular joint disturbance syndrome  treatment of tmj  mandibular dysfunction  significant improvement in tmj pain  temporomandibular joint involvement  bilateral temporomandibular joint hypermobility referred  temporomandibular joint arthroscopy  tmj internal derangement  management of condylar resorption  tmj hypermobility  temporomandibular joint ankylosis from  tmj tumor defects  temporomandibular joint ankylosis poses anterior displaced temporomandibular discs with unstable excursive reduction  displaced temporo- mandibular disc syndrome  mandibular disc syndrome  temporomandibular joint disk displacement  tmj disk displacement  type of temporomandibular joint disk displacement  temporomandibular joint anterior disk displacement  temporomandibular joint anterior disk displacement without reduction  mandibular condylar process fractures  unilateral mandibular condylar process fractures  tmj symptoms  mandibular condyle fractures  surgical treatment of mandibular condyle fractures  temporomandibular joint arthrocentesis post-traumatic tmd  trauma-induced tmd  temporomandibular joint pain-dysfunction syndrome  temporomandibular joints with reciprocal clicking  treatment of temporomandibular joints  fracture of the mandibular condyls  chronic closed lock  temporomandibular joint disc repositioning  tmj articular disc repositioning  tmj disc repositioning  trigger points in masticatory muscles  temporomandibular myofascial pain  treatment of temporomandibular myofascial pain craniofacial myalgia  patients with craniofacial myalgia  painful temporomandibular joints  treatment of painful temporomandibular joints  temporomandibular joint interal derangement  tmjid  displaced temporomandibular joint meniscus  temporomandibular joints with anterior meniscus displacement  mandibular sub-condylar fractures  sub-condylar fractures  sub-condylar mandibular fractures  subcondylar mandibular fracture | tmj disc histopathology  active bilateral idiopathic condylar resorption treated  myofascial pain symptoms in bruxers  chronic tmj disease  temporomandibular joint myofascial pain  treatment of temporomandibular joint ankylosis  treating tmj ankylosis  temporomandibular joint ankylosis treated  modified temporalis anchorage in craniomandibular reankylosis  progressive condylar resorption  myogenic cranio-cervical-mandibular dysfunction  development of condylar resorption  craniomandibular disorder  temporomandibular muscle pain dysfunction  tmjds  temporomandibular joint involvement in childhood arthritis  outcome variables of tmj hypermobility  temporomandibular joint arthroscopy improve outcomes  postoperative jaw opening exercises  treatment of temporomandibular joint internal derangement  post surgical tmj medical management  tmj pain  tmj ankylosis in delta nile  tmj internal derangement suggests  active idiopathic condylar resorption  detection of tmj disease  tmd clinic  treatment of tmj ankylosis  tmj re-ankylosis  severe jaw muscle pain  reducing masseter muscle pain  progressive condylar resorption following orthognathic surgery  subject of condylar resorption  craniomandibular disorder with respect  treat acute temporomandibular muscle pain dysfunction  temporomandibular joint hypermobility treatment  tmj capsular width  wilkes stage iv  wilkes stage  study selection included preexisting cbct condylar resorption  tmj pain on palpation  temporomandibular joint ankylosis surgical treatment  treatment modalities of tmj ankylosis  active idiopathic condylar resorption treated  high prevalence of temporomandibular joint arthritis  tmj mp  post-operative condylar resorption  temporo-mandibular-joint complaints  tmj inflammation  temporomandibular joint hypermobility?  wilkes stage iv internal derangement  surgical tmj  treatment of symptomatic tmj hypermobility  various types of tmj ankylosis  bilateral condylectomy  treatment of temporomandibular joint myofascial pain  preoperative condylar resorption  tolerable temporo-mandibular-joint complaints  tmj hypermobility treatment  wilkes stage iv internal derangement?  tmj ankylosis seeking treatment  wilkes stage iv from  treatment of temporomandibular joint hypermobility  treatment of temporomandibular joint myofascial pain  myogenous cranio-mandibular disorder  tmj locking  tmj hypermobility assessed  wilkes stage iv presenting  verbal scale expressing tmj pain  prevalence of temporomandibular joint disease  simple type of myogenous cranio-mandibular disorder  tmj pain intensity  tmj prolotherapy  wilkes stages  wilkes stages ii  wilkes stages ii through v  signs of tmj involvement  tmj arthritis  tmj involvement  temporomandibular joint disease  tmj disease  research diagnostic criteria for temporomandibular disorders diagnosis  rdctmd  temporomandibular joint internal derangements  treatment of temporomandibular joint internal derangements  chronic tmj degenerative disorders  management of chronic tmj degenerative disorders  temporomandibular joint degenerative disorders  temporomandibular disorder cases  temporomandibular joint internal derangements assessed  jaw muscle pain reduction  clinical pain among myofascial temporomandibular disorder participants  cohort of myofascial temporomandibular disorder participants  myofascial temporomandibular disorder participants  temporomandibular disorder participants  chronic tmjd pain  clinically significant tmjd pain  progression of chronic tmjd pain  significant tmjd pain  tmjd  tmjd chronicity  tmjd pain  chronic recurrent temporomandibular joint dislocation  chronic recurrent tmj dislocation  recurrent tmj dislocation  temporomandibular joint dislocation tmj for treatment  treatment of chronic recurrent tmj dislocation  recurrent temporomandibular joint dislocation  temporomandibular joint dislocation  treatments for recurrent temporomandibular joint dislocation  mandibular condylar fractures  temporomandibular joints with closed treatment  treatment of mandibular condylar fractures  chronic tmd pain  temporomandibular dysfunction pain  mandibular subcondylar fractures  maxillomandibular fixation for subcondylar fractures  treatment of mandibular subcondylar fractures  anterior displaced temporo anterior displaced temporomandibular discs  anterior displaced temporomandibular discs with unstable excursive reduction  displaced temporo- mandibular disc syndrome  mandibular disc syndrome  temporomandibular joint disk displacement  tmj disk displacement  type of temporomandibular joint disk displacement  temporomandibular joint anterior disk displacement  temporomandibular joint anterior disk displacement without reduction  mandibular condylar process fractures  unilateral mandibular condylar process fractures  tmj symptoms  mandibular condyle fractures  surgical treatment of mandibular condyle fractures  temporomandibular joint arthrocentesis  temporomandibular joint arthrocentesis for acute  tmj arthrocentesis  tmj procedures  nonreducing temporomandibular joint disk displacement  temporomandibular joint arthrography  temporomandibular joint arthrography alone  temporomandibular joint osteoarthrosis  tmj osteoarthrosis  classification of temporomandibular joint osteoarthrosis  temporomandibular joint syndrome  tmj for treatment  anterior displaced temporomandibular discs  temporomandibular joint effusion  tmj effusion  subcondylar fractures of the mandible  arthropathy of the temporomandibular joint in children  cervico-craniofacial pain  cervico-craniofacial pain of myofascial origin  temporomandibular disorder signs  temporomandibular signs  tmd  tmd signs  disk displacement with reduction  temporomandibular disk displacement  temporomandibular disk displacement with reduction  temporomandibular joint clicking  tmj clicking  bilateral temporomandibular joint symptomatology  presence of significant temporomandibular joint pathology  significant temporomandibular joint pathology  temporomandibular joint pathology  temporomandibular joint symptomatology  tmj intracapsular pain  tmj intracapsular pain according  temporomandibular joint symptoms  temporomandibular disorder pain dysfunction syndrome  temporomandibular disorder pain dysfunction syndrome affect dietary intake?  arthritic mandibular condyles |
| --- | --- | --- |
